# IL-15 and IL15RA in Osteoarthritis: Association With Symptoms and Protease Production, but Not Structural Severity

**DOI:** 10.3389/fimmu.2020.01385

**Published:** 2020-07-23

**Authors:** Sophie C. Warner, Anjali Nair, Rahul Marpadga, Susan Chubinskaya, Michael Doherty, Ana M. Valdes, Carla R. Scanzello

**Affiliations:** ^1^Academic Rheumatology, Nottingham City Hospital, Nottingham, United Kingdom; ^2^Section of Rheumatology, Rush University Medical Center, Chicago, IL, United States; ^3^Division of Pediatrics, Rush University Medical Center, Chicago, IL, United States; ^4^Arthritis Research UK Pain Centre and National Institutes for Health Research Nottingham Biomedical Research Centre, Nottingham, United Kingdom; ^5^Translational Musculoskeletal Research Center & Section of Rheumatology, Corporal Michael J. Crescenz VA Medical Center, Philadelphia, PA, United States; ^6^Division of Rheumatology, University of Pennsylvania Perelman School of Medicine, Philadelphia, PA, United States

**Keywords:** inflammation, pain, proteases, interleukins, interleukin-15, osteoarthritis, neuropathic pain, neuropathic pain-like symptoms

## Abstract

**Objective:** Interleukin-15 (IL-15) is a pro-inflammatory cytokine that is increased in joint fluids of early-stage osteoarthritis (OA) patients, and has been associated with expression of proteases that can damage cartilage, and the development of neuropathic pain-like symptoms (NP) after nerve injury. The objective of this study was to further explore the role of IL-15 in the pathogenesis of OA cartilage degeneration and test genetic variation in the IL-15 receptor α gene (*IL15RA*) for an association with OA with radiographic severity and symptoms.

**Methods:** Cartilage samples from donors (*n* = 10) were analyzed for expression of the IL15 receptor α-chain using immunohistochemistry, and for responses to IL-15 *in vitro* using explant cultures. Data from two independent Nottinghamshire-based studies (*n* = 795 and *n* = 613) were used to test genetic variants in the *IL15RA* gene (rs2228059 and rs7097780) for an association with radiographic severity, symptomatic vs. asymptomatic OA and NP.

**Results:** IL-15Rα was expressed in chondrocytes from cartilage obtained from normal and degenerative knees. IL-15 significantly increased the release of matrix metalloproteinase-1 and -3 (MMP-1 and -3), but did not affect loss of proteoglycan from the articular matrix. Genetic variants in the *IL15RA* gene are associated with risk of symptomatic vs. asymptomatic OA (rs7097780 OR = 1.48 95% 1.10–1.98 *p* < 0.01) and with the risk of NP post-total joint replacement (rs2228059 OR = 0.76 95% 0.63–0.92 *p* < 0.01) but not with radiographic severity.

**Conclusions:** In two different cohorts of patients, we show an association between genetic variation at the IL15 receptor and pain. Although *ex vivo* cartilage explants could respond to IL-15 with increased protease production, we found no effect of IL-15 on cartilage matrix loss and no association between *IL15RA* variants and radiographic severity. Together, these results suggest that IL-15 signaling may be a target for pain, but may not impact structural progression, in OA.

## Introduction

The inflammatory response has been shown to play an important role in osteoarthritis (OA) (1), both in symptomatic and structural manifestations, but the relative importance of specific inflammatory mediators in OA joints is yet to be fully elucidated. Previous work has evaluated common-gamma chain cytokines and specifically detected interleukin-15 (IL-15) in many synovial fluid (SF) specimens from people with knee OA (2, 3). IL-15 is a pro-inflammatory cytokine that is best characterized for its effects on T-lymphocyte and NK-cell activation, proliferation, and survival (4, 5). We previously found IL-15 levels in the SF to be elevated in early-stage disease compared with advanced disease and correlated with SF matrix metalloproteinases-1 and -3 (MMP-1 and MMP-3) levels (2). These two proteases are often elevated in the joints of OA patients and have been linked to extracellular matrix turnover. Using a proteomic approach to identify biomarkers of disease, other investigators found IL-15 detectable in serum samples from participants in the Baltimore Longitudinal Study of Aging (BLSA) (6). In this study, serum IL-15 was associated with radiographic OA changes in the knee and hands, and was elevated in individuals up to 10 years prior to observation of radiographic changes. Taken together, these studies suggest that IL-15 might play a role in early initiation events leading to joint degeneration in OA.

IL-15 has also been linked with pain in various rheumatologic diseases including rheumatoid arthritis (RA), lupus, and OA (2, 7–11). IL-15 inhibition has been tested in clinical trials for RA, and has been demonstrated to improve pain (12). In addition to nociceptive pain, IL-15 has been linked to the development of neuropathic pain-like symptoms (NP) after nerve injury, due to its role in neuroinflammation (13). In OA, multiple pain phenotypes have been described including NP (14, 15) and neuroinflammatory mechanisms have been implicated mechanistically [reviewed in (16)].

The association of IL-15 with the emergence of radiographic changes, protease production within the joint, and pain severity in OA, suggest that this cytokine may play multiple roles in OA disease pathogenesis. Importantly, IL-15 signaling is dependent on binding to the high specificity IL-15 receptor chain, interleukin-15 receptor α (IL-15Rα), encoded in humans by the *IL15RA* gene (17). The aims of this study therefore were to explore the potential effects of IL-15 on OA by examining both structural and symptomatic disease manifestations. Given the association with protease production in previous studies, we first evaluated whether chondrocytes express IL-15Rα, and whether IL-15 has a direct effect on protease production and matrix loss from human cartilage *in vitro*. We then investigated whether select *IL15RA* variants were associated with symptoms or radiographic severity in OA by examining two separate cohorts of patients.

## Materials and Methods

### *In vitro* Cartilage Experiments

#### Cartilage Donors

**Figure 1:**
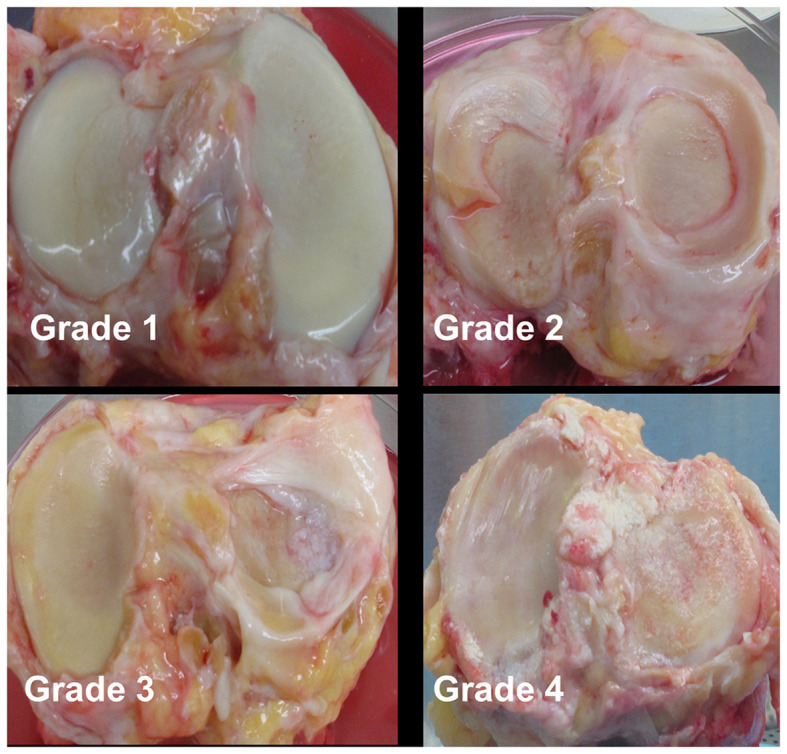
Representative gross morphology of donor knees. Grade 1–4 represent modified Collins grade as follows: Grade 1 = limited disruption of the articular surface with only minor fibrillations; Grade 2 = fibrillation of cartilage with fissures ± small osteophytes; Grade 3 = osteophytes + cartilage fibrillation and fissuring with 30% or less of the cartilage surface eroded down to subchondral bone; Grade 4 = osteophytes and gross geometric bony change + >30% of the cartilage surface eroded down to the subchondral bone.

Cartilage was collected within 24-h post-mortem from the knees of ten organ donors with no history of arthritis through the Gift of Hope Organ and Tissue Donor Network (Itasca, IL), through an IRB-approved bio-repository at Rush University Medical Center. Age and gender of the donors was recorded. Degenerative changes in the knee joint were evaluated by a pathologist at the time of dissection, according to the modified Collins grade (18). When degenerative changes were present, cartilage was taken from lesional areas. The characteristics of these donors are presented in Table 1, and representative gross morphology presented in Figure 1.

**Table 1:** Characteristics of cartilage tissue specimens used for *in vitro* experiments.

**Donor**	**Age range**	**Collins grade^**#**^**	**Cartilage characteristics**
1	18–25	0	Normal
2	46–50	1	Minor fibrillation
3^*^	66–70	1	Minor fibrillation
4	71–75	1	Minor fibrillation
5	51–55	2	Fibrillation + fissures
6	61–65	2	Fibrillation + fissures
7	71–75	2	Fibrillation + fissures
8	66–70	3	≤30% full-thickness erosion
9	66–70	3	≤30% full-thickness erosion
10	56–60	4	≥30% full-thickness erosion

* *Femoral cartilage was obtained from specimen #3. Tibial cartilage was obtained from all other donors*.

#### Immunohistochemistry

Tibial cartilage specimens from three organ donors were formalin-fixed and paraffin-embedded. Six-micron sections were prepared and stained for IL-15Rα using standard immunoperoxidase technique and a polyclonal antibody directed against the human IL-15Rα chain (goat anti-human IL-15Rα, Santa Cruz Biotechnology). Non-immune goat IgG was utilized as negative control for staining specificity.

#### Explant Culture

To test effects of IL-15 on cartilage, 4 mm cartilage punch biopsies were prepared from the tibial or femoral surfaces of organ donors (*n* = 10 donor experiments, each run in duplicate, Table 1). For each donor, 2 explants per well were placed in a 24 well plate with 1 ml DMEM (+100 U/ml Penicillin-Streptomycin). After 24 h, media was replaced with 1 ml of fresh media with or without recombinant human IL-15 (100 ng/ml, Peprotech, NJ). The concentration of IL-15 was chosen based on the range of concentrations required for *in vitro* stimulation of NK cell proliferation and activation (19). TNF-α + Oncostatin M (100 ng/ml each, R & D Systems, MN), a potent stimulus for MMP production and cartilage proteoglycan loss (20, 21) was used as a positive control. Every 2 days for up to 10 days, culture supernatants were collected and replaced with fresh media with or without cytokines. Consecutive 2-days supernatants were analyzed in duplicate from each well, and two wells per donor analyzed, for MMP-1, -3, and -9 using a human MMP 3-plex ultrasensitive electrically-activated chemiluminescence immunoassay (Meso Scale Discovery, Rockville MD) read on a Sector 6000 Imager. Total ng in each supernatant aliquot was determined, cumulative amounts at each time point calculated, and results were expressed as the percentage of the total ng released in 10 days from the untreated control (=100%) to allow comparison between donors.

Explant supernatants (*n* = 7 donors) were processed for measurement of glycosaminoglycan (GAG) release using the Dimethyl Methylene Blue (DMMB) assay (22). For three donors, total GAG content (explants + supernatants) was measured as follows. Cartilage explants (fresh day 0 and those after 14 days of culture) were extracted and digested according to previously reported methods (23). Extractable GAG content of the explants was then determined, and total extractable GAG content (the sum of supernatants up to 14 days + explants after 14 days) was calculated and compared to unstimulated cultures. The percentage of the total GAG content released into the media was calculated as a measure of GAG breakdown.

### Genetic Study of IL-15 Receptor Variants

#### Study Participants

A subset of knee OA cases from the Nottinghamshire-based Genetics of OA and Lifestyle (GOAL) study and from the Nottingham Genetics Case Control study [previously described (24)] were used in this analysis to assess the role of *IL15RA* variation on radiographic severity and symptoms in OA. All individuals from both cohorts had X-rays assessed at baseline. Analyses here are based on tibiofemoral (TF) Kellgren/Lawrence (K/L) grade. Symptomatic or asymptomatic status was available only for participants in the GOAL study. Additionally, hip OA cases are not included here because of a lack of asymptomatic hip OA cases. Individuals classified as asymptomatic OA cases were originally recruited as controls for OA and were later found to have radiographic evidence of knee OA, despite not reporting knee pain.

A postal questionnaire about joint pain, quality of life and medical history was sent on average 4.8 years later to individuals from both cohorts, including hip OA cases not included in the knee OA study, when most (91.4%) individuals had undergone a total joint replacement (TJR).

The North Nottinghamshire Research Ethics Committee gave approval for the ethics of the two studies. All participants gave written, informed consent (according to the Declaration of Helsinki).

#### Binary Trait Definitions for Statistical Analysis

The joint-specific version of the painDETECT questionnaire (PDQ) for NP symptoms of the joint was used (25), and included in the postal questionnaire administered after enrollment. Possible joint NP was classified as a score of >12 on the site-PDQ as described and validated previously (25). TF K/L scores were dichotomized as 0 = grade 2, 1 = grades 3 and 4 (in OA-affected individuals in both groups).

#### Genetic Data

Blood samples from the participants in this study were processed to obtain genotype data as previously described (26). These data were available for all participants in this part of the study. Binary logistic regression analysis was used to test two genetic variants (SNPs) in the *IL15RA* gene, rs2228059 (mapping to chromosome 10 position 5960405, minor allele, C, with a frequency of 0.492) and rs7097780 (mapping to chromosome 10 position 5968264, minor allele G with a frequency of 0.323) for an association with TF K/L grade of radiographic severity and possible NP in both groups. These two SNPs together represent 67% of the variation in the *IL15RA* gene (https://snpinfo.niehs.nih.gov/snpinfo/snptag.htm).

Symptomatic vs. asymptomatic OA variables were also tested in the GOAL group only. The statistics package R (version 3.0.2) was used for these analyses. All analyses were adjusted for age, sex, and BMI. Meta-analysis was used to test the overall effect of *IL15RA* genotype across both groups where necessary.

## Results

### Articular Chondrocytes Express the IL-15 Receptor α-Chain

**Figure 2:**
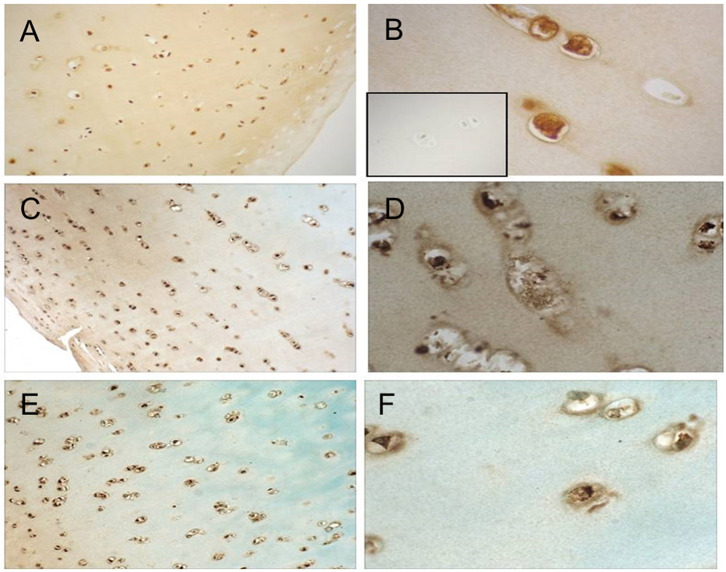
IL-15Ra staining in human articular cartilage. Cartilage specimens were processed and immunostained as described. Representative photomicrographs of cartilage from a grade 0 donor **(A,B)**, grade 2 donor **(C,D)**, and grade 3 donor **(E,F)** at 10× **(A,C,E)** and 40× **(B,D,F)** are shown. Isotype-matched negative control staining is shown in inset of **(B)**.

Cellular responses to IL-15 are mediated by binding to the IL-15 receptor which is composed of three subunits: the IL-15Rα chain which confers binding specificity for IL-15, and a common β and γ chain shared by the IL-2 receptor (17). We therefore studied expression of IL-15Rα by articular chondrocytes. Formalin fixed, paraffin-embedded articular cartilage specimens from three donors (one each of grade 0, 2, and 3) were sectioned for immunohistochemical staining as described. As depicted in Figure 2, staining for IL-15Rα was observed in chondrocytes regardless of Collins grade. In addition, staining was observed throughout the thickness of the cartilage, from the superficial layer to the deep zone.

### IL-15 Induces MMP-1 and MMP-3 Production From Articular Cartilage *in vitro*

**Figure 3:**
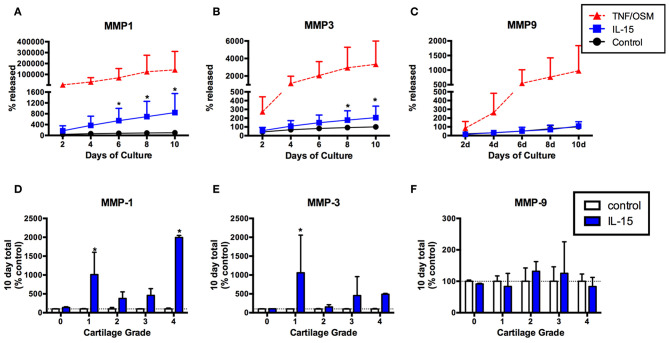
IL-15 effect on MMP-1, -3, and -9 release from human cartilage explants over 10 days *in vitro*. Articular cartilage explants were exposed to IL-15 (100/ml) *in vitro* for 10 days as described, and supernatants collected and replaced every 2 days. Explants exposed to TNF + OSM served as a positive control. MMP-1, -3, and -9 were measured in explant supernatants at each time point, and levels expressed as the percent of the total amount produced in 10 days in unstimulated control cultures (mean ± SD of 10 experiments). IL-15 increased release of **(A)** MMP-1 and **(B)** MMP-3 compared to unstimulated control explants (*p < 0.001, 2-way ANOVA). **(C)** No effect of IL-15 on MMP-9 release was observed. **(D,E)** The effects of IL-15 on **(D)** MMP-1 and **(E)** MMP-3 release was highly variable, with the most significant differences observed in explants from donors with early (grade 1) degenerative changes. **(F)** There was no release of MMP-9 in response to IL-15, regardless of explant grade.

Given the association of IL-15 with protease activity (3) we next tested whether IL-15 exposure could induce MMP production from articular chondrocytes using an *in vitro* explant system. MMP-1, -3, and -9 production was measured in explant supernatants collected every 2 days for 10 days. Levels were expressed as the percentage of the total released in 10 days without cytokine stimuli (100%). Release of MMP-1 in response to IL-15 was observed in cartilage explants from eight of 10 donors (mean ± SEM MMP-1 release: 843% ± 222 of control, Figure 3A), and MMP-3 in five of 10 donors (mean MMP-3: 206% ± 42 of control, Figure 3B). This release was delayed (observed at 4–6 days) compared to the positive control stimuli (TNF/OSM). IL-15 had no effect on MMP-9 release (mean 109% ± 17 of control, Figures 3C,F). When examined according to Collins grade of the explant donor, MMP-1 release in response to IL-15 treatment was observed in explants with grade 1 through 4 cartilage degeneration, but not in the normal (grade 0) cartilage donor (Figure 3D). A similar pattern was observed with MMP-3 (Figure 3E).

As expected (20, 21), robust but variable MMP-1 and MMP-3 production was observed in response to the positive control stimuli TNF + OSM from all explants, whether from normal or degenerative donors (Supplemental Figures 1A,B). We found no significant differences in the TNF + OSM response when comparing donors that responded to IL-15 vs. non-responders (Supplemental Figures 1C,D, Mann-Whitney *P* > 0.05), and no statistically significant correlation between the responses to IL-15 and TNF + OSM, for either MMP-1 (Spearman *r* = 0.54, *P* = 0.11) or MMP-3 (*r* = 0.05, *p* = 0.89) (Supplemental Figures 1E,F).

### IL-15 Does Not Promote GAG Loss From Articular Cartilage

**Figure 4:**
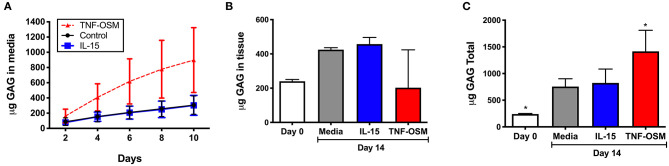
Effect of IL-15 on GAG release and tissue GAG content *in vitro*. **(A)** Cartilage explants (*n* = 7 experiments) were exposed to 100 ng/ml rhIL-15 (blue squares) or left unstimulated (control black circles) as described. Exposure to TNFα and Oncostatin M (TNF-OSM, 100 ng/ml each, red triangles) served as a positive control for proteoglycan release. The cumulative GAG content of the media collected every 2 days for up to 10 days is depicted over 10 days in culture. IL-15 had no significant effect on GAG loss from explants compared to unstimulated control cultures. **(B)** GAG content of cartilage explants (*n* = 3 separate experiments) collected at day 0 (fresh) and after 14 days of culture was measured as described. IL-15 had no effect on cartilage tissue GAG content after 14 days compared to control unstimulated explants. TNF/OSM led to net loss (*p* = ns) of cartilage GAG content as expected. **(C)** The total GAG content of the culture system (explants + media aliquots) after 14 days *in vitro* was calculated to evaluate GAG synthesis and subsequent release. Total GAG content was increased at 14 days compared to baseline in unstimulated controls, indicating a net synthesis of GAGs in the explants. This was not further impacted by exposure to IL-15. *p < 0.05 compared to day 14 unstimulated explants.

GAG content of culture supernatants was measured in seven experiments, to determine whether IL-15 impacted GAG loss from cartilage matrix. Over 10 days in culture there was an average loss of 306 (±46) μg GAG from the unstimulated explants that was not further enhanced by exposure to IL-15 (303 ± 50 μg, Figure 4A). Explants responded as expected to TNF/OSM with a loss of 897 (±161) μg in 10 days. Since cytokines can also modulate GAG synthesis by chondrocytes, we measured GAG content in explants from 3 of the donors as described in **Methods**. As shown in Figure 4B, over a 14 days culture period, GAG content increased slightly in unstimulated explants (240.0 ± 6.0 μg at day 0, to 424.8 ± 6.7 μg at day 14) suggesting some new synthesis. Exposure to IL-15 did not alter this increase (457.3 ± 22.4 μg). In contrast, in 2 of 3 experiments there appeared to be a decrease of GAG content of the tissue compared to day 0 explants in response to TNF/OSM exposure, although mean explant GAG was not different from unstimulated controls at day 14 (202.6 ± 127.6 μg, Figure 4B). When total GAG content of the system (14 days tissue + supernatants) was calculated there was no difference in unstimulated and IL-15 stimulated groups, while TNF/OSM exposure led to a net increase in total GAG content (media + explant) reflecting both enhanced synthesis and subsequent release into the media (Figure 4C).

### Genetic Variation in the IL-15 Receptor Is Associated With Symptomatic OA and With Neuropathic Pain-Like Symptoms but Not With Radiographic Severity

The effects of IL-15 are dependent on binding to its highly specific receptor (17) which is encoded by the *IL15RA* gene. We next used a genetic approach to assess if there is a relation between *IL15RA* variation, radiographic severity and pain measures in people with OA. The descriptive characteristics of the participants with knee OA and post-TJR in this genetic study are shown in Table 2.

**Table 2:** Descriptive statistics for the groups used in the genetic analysis.

	**GOAL study**	**Nottingham genetics**
		**of OA study**
Tibiofemoral OA cases *n* (%F)	551 (48.3%)	403 (54.8%)
Age (SD)	66.6 (6.7)	69.3 (8.8)
BMI (SD)	30.1 (5.0)	29.8 (5.1)
TF K/L grade (2/3/4)	116/295/140	30/261/112
Symptomatic/Asymptomatic TF OA	403/148	403/0
Post-TJR cases n	795	613
(F%, % post-total knee replacement)	(48.1%. 53.6%)	(56.8%, 64.9%)
painDETECT score mean (SD)	4.49 (6.3)	4.99 (7.6)
Possible neuropathic pain (%)	152 (13.8%)	109 (17.8%)
Unlikely neuropathic pain (%)	948 (86.2%)	504 (82.2%)
rs2228059 (C/A)	0.508/0.492	0.502/0.498
rs7097780 (G/A)	0.677/0.323	0.660/0.340

We found no significant association between either of the *IL15RA* variants and radiographic severity of knee OA (Table 3), although there was a trend between the G allele frequency at rs7097780 and TF K/L grade (K/L = 2: 30.2%, K/L = 3: 33.3%, K/L = 4: 36.2%, Armitage trend test *p* = 0.07). On the other hand, the frequency of the G allele was associated with risk of OA symptoms. A significant association was seen between the rs7097780 genotype and the risk of symptomatic vs. asymptomatic knee OA. The unadjusted analysis gave a result of: OR = 1.56 (1.18–2.07), *p* = 0.0019. After adjusting for age, sex and BMI the odds ratio remained significant, at 1.48 (1.10–1.98), *p* = 0.0098 (Table 3). Further adjustment for maximum TF knee OA grade gave a result of: OR = 1.43 (1.04–1.99), *p* = 0.029. To determine whether results could be driven by the subgroup of patients with NP, we re-ran the analysis removing participants with possible NP from the model. This reanalysis demonstrated an OR = 1.45 (1.05–2.01), *p* = 0.025.

**Table 3:** Association between knee OA and pain-related traits and two *IL15RA* SNPs.

**Trait**	***IL15RA*** **SNP**	
	**rs2228059 (effect allele = C)**	**rs7097780 (effect allele = G)**
Radiographic severity: K/L grade 2 vs. grades 3 + 4	OR = 0.98 (0.75–1.27)	OR = 1.19 (0.90–1.57)
Symptomatic vs. asymptomatic knee OA	OR = 1.02 (0.76–1.36)	**OR = 1.48 (1.10–1.98)**, ***p*** **= 0.0098**
Neuropathic pain post-TJR	**OR = 0.76 (0.63–0.92)**, ***p*** **= 0.005**	OR = 0.94 (0.76–1.15)

*Odds ratios (OR) are adjusted for age, sex, and BMI. Bold values indicate statistically significant results (p < 0.01)*.

The minor (C) allele in rs2228059 was associated with a lower risk of NP joint symptoms post-TJR. Before adjusting for covariates, the effect of rs2228059 genotype on the risk of NP in the GOAL cohort was: OR = 0.75 (0.58–0.96). A very similar effect size is seen in the Nottingham replication cohort: OR = 0.81 (0.60–1.09). After adjustment for covariates and meta-analysis of both cohorts, this achieves: OR = 0.76 (0.63–0.92), *p* = 0.005 (Table 3).

## Discussion

In this study, we explored the previously reported associations between IL-15, protease production, and pain in knee OA patients (3). Our results show associations of IL15RA variants with symptoms in knee OA, supporting a role for IL-15 signaling in symptomatic manifestations in patients with established OA. In contrast, we found no evidence to support a role for IL-15 in promoting structural damage, as there was no association with radiographic severity in the cohort studies, and IL-15 did not stimulate cartilage degradation despite promoting MMP production in the explants.

Given our previous finding of an association between IL-15, MMP-1, and MMP-3 concentrations in patient synovial fluids (3), we began by testing whether IL-15 could directly impact cartilage protease production *in vitro* after confirming IL-15 receptor expression by human chondrocytes (Figure 2). Exposure of human articular cartilage to rhIL-15 led to cartilage secretion of MMP-1 and -3, but not MMP-9 (Figure 3), validating our previous results. These proteases are commonly observed to be upregulated in the setting of joint injury and arthritis (27). Although not as potent a stimulus as our positive control (TNFα + OSM), IL-15 induced measurable release of MMP-1 from 8 of 10, and MMP-3 secretion from 5 of 10 donors tested. Average (±SEM) MMP-1 release was 843 (±222)% and MMP-3 release 206 (±42)% greater than in unstimulated explants. The response to IL-15 was delayed compared to the positive control, raising the possibility that effects of IL-15 are indirect. IL-15 is known to induce production of other cytokines including TNFα (28) which could explain a delayed effect. Whether IL-15 could additionally synergize with TNFα to further promote MMP activity needs further exploration. In this study, we found no correlation between the responses of explants to TNF + OSM and IL-15 (Supplemental Figure 1), although it is possible we were underpowered to detect a subtle relationship. Still, to our knowledge this is the first report that this cytokine can directly activate human articular chondrocytes. Interestingly, protease production above control levels was not observed from the normal cartilage specimen, despite IL-15Rα expression. The small number of specimens utilized in this study precludes conclusions as to whether the impact of IL-15 on chondrocytes is dependent on age or the presence of degenerative changes, which will need to be tested in larger studies. But the response clearly requires additional factors beyond IL-15Rα expression. Moreover, these experiments demonstrate the importance of utilizing multiple tissue donors to assess human cartilage activity in explant culture systems, given the inherent variability of the response.

Despite our observation that IL-15 could induce MMP-1 and -3 production from cartilage explants *ex vivo*, this did not result in matrix loss and we found no association of *IL15RA* genetic variation with radiographic severity in the OA cohorts. This suggests that IL-15 likely does not play an important role in progression of cartilage degeneration in OA. Articular cartilage degradation in arthritic diseases involves a complex milieu of proteases that can cleave the proteoglycan and collagen networks of the articular matrix, leading to progressive matrix loss. Members of the MMP, cathepsin, and ADAMTS enzyme families cleave aggrecan (the main proteoglycan of the articular matrix) at multiple sites leading to an array of fragments or “neoepitopes” that are detectable in joint fluids and cartilage (29). In mice, evidence suggests that the aggrecanase ADAMTS5 is responsible for cleavage events leading to aggrecan loss from the matrix, but in humans multiple enzymes including ADAMTS-4,−5 and a variety of MMPs are thought to contribute (30). MMP-3 in particular is thought to play a role in progression of structural disease (31). Given our observed effect of IL-15 on protease production including MMP-3, we proceeded to test release of GAG fragments (as a measure of aggrecan breakdown) from the cartilage explants exposed to IL-15. We saw no release above unstimulated levels (Figure 4). In addition, no effect on aggrecan tissue content or net loss from the matrix was observed. We did not assess protease activity directly in this study, so it is possible that IL-15 increases enzyme production, but activity is controlled by molecular inhibitors (i.e., TIMPs, or tissue inhibitors of metalloproteinases) and other mechanisms. However, our results are consistent with recent evidence demonstrating that MMPs may be more involved in aggrecan turnover events within the matrix rather than aggrecan depletion from the matrix (32).

As we did not find that IL-15 on its own was directly pathogenic to articular cartilage, we proceeded to investigate a relationship with symptoms in OA, given reports of associations between IL-15 and pain in rheumatologic diseases (2, 7–11). Using genetic data available from the GOAL study we found that a variant in the *IL15RA* gene is associated with higher risk of symptomatic OA, but not with radiographic severity. Specifically, the presence of the G allele of the rs7097780 IL15RA variant was associated with a 1.48 (1.10–1.98)-fold higher risk of symptoms in patients with definite radiographic changes, and this risk was not affected by the severity of radiographic findings. In addition, removing the patients with possible NP from the analysis did not change the results significantly. Although it is unclear whether these results are related to the effect of IL-15 on protease production seen in the cartilage explants, it is possible that effects on pain may also be indirect. MMP mediated processing is implicated in the generation of a small peptide fragment of the aggrecan core protein (33) which can mediate inflammatory signaling in multiple cell types (34) and has been linked to pain-generation in OA models (35). Additionally, cartilage may be a source of molecular mediators of OA pain, such as nerve growth factor and tachykinin, as recently demonstrated in animal models (36). Independent of its effect on protease production, IL-15 may have direct effects on nociception. When injected into the footpads of mice (37), or intra-thecally in rats (38), IL-15 augmented pain-related outcomes. In this report, although we demonstrate that cartilage expresses the specific IL-15 receptor and can respond biologically to IL-15, whether cartilage itself is a source of IL-15 in the joint, or IL-15 induces other nociceptive mediators from cartilage, remains to be tested.

Another novel finding of this study is the relationship between the rs2228059 minor (C) allele and protection from NP post-TJR. This was demonstrated using data available from both cohorts of patients. However, this is unlikely to be related to the effect of IL-15 on cartilage as it was observed in individuals post-TJR, after native cartilage has been removed. It has been suggested that NP may be improved by inhibition of IL-15 (39). A proportion of individuals with OA suffer from NP (40, 41) so this cytokine may prove to have a relevant role in both nociceptive and neuropathic pain in OA. IL-15 can regulate inflammatory cell infiltration locally and in the peripheral nervous system after nerve injury, linking inflammation and pain generation (13, 39). Our data suggests here is a role to be investigated in more detail for IL-15 in joint NP pain that is not dependent on its effects on cartilage.

The link between IL-15 levels and radiographic severity is still unclear (2). A previous report demonstrated a relationship between serum IL-15 levels and emergence of radiographic changes (6). In the current genetic association study we found no consistent effects of *IL15RA* genotype on radiographic severity in two independent patient cohorts, after adjusting for common confounders. In both the GOAL and Nottingham study, only patients with K-L radiographic stage 2 or greater were examined, as opposed to the earlier study in which patients with no X-ray abnormalities at baseline were followed. Therefore, it is possible that we may have seen an effect of these genetic variants if patients with earlier stage disease were examined. However, current results together with our observation that IL-15 had no clear effect on cartilage GAG loss, suggest that the role of this cytokine in OA may be primarily on symptomatic manifestations of disease.

There are a number of limitations to the current study that need to be considered. For the *in vitro* studies we were only able to obtain normal cartilage from a single donor, limiting our ability to determine whether variability in the IL-15 response was related to the presence of degenerative changes. In addition, our assays did not distinguish between active and total MMP concentrations. With regards to the genetic study, we were able to see the same effect on NP in the two cohorts from the same recruitment area, but the lack of asymptomatic cases in the second cohort prevents us from validating our association with symptoms. Moreover, the lack of information on NP prior to joint replacement in the second cohort limits our analysis of NP at earlier stages of disease. Thus, these results require further replication. In addition, neither of the variants tested has a known functional role, e.g., in terms of ligand binding or expression, so we cannot directly link the genetic association seen to increased or decreased IL-15.

This is the first demonstration that cartilage is responsive to IL-15. Our data suggests a novel role for IL-15 in promoting protease release from cartilage, consistent with previous reports showing associations between IL-15 and protease levels in OA patients (3, 42), but the significance of this effect is as yet unclear as no effect on cartilage matrix loss was seen. As protease levels, particularly MMP-3 levels are commonly elevated in patient synovial fluids, it is possible that these proteases are a reflection of underlying inflammatory activity driven by cytokines, such as IL-15. IL-15Rα blockade can reduce acute inflammation (43), and joint inflammation specifically in an animal model of inflammatory arthritis (44). The association we found between specific *IL15RA* SNPs and symptoms in OA is particularly novel and interesting, as an association between IL-15 levels and OA pain has been reported (2). However, the impact of the specific *IL15RA* alleles studied on IL-15 signaling has not yet been well-studied. More research is needed to identify the effects of these genetic variations on IL-15 activity. But the results of this analysis support previous findings that IL-15 or IL-15 related activity may potentially be a biomarker to help assess disease severity in OA (2). Whether IL-15 activity or levels predict emergence of peripheral or central sensitization in the generation of neuropathic-type pain in OA is an important question that will also need to be addressed in future, longitudinal studies.

## Data Availability Statement

The raw data supporting the conclusions of this article will be made available by the authors, without undue reservation, to any qualified researcher.

## Ethics Statement

The studies involving human participants were reviewed and approved by Rush University Medical Center Institutional Review Board and the North Nottinghamshire Research Ethics Committee. The patients/participants provided their written informed consent to participate in this study.

## Author Contributions

SW, AV, and CS: substantial contributions to the conception or design of the work. SW, AN, RM, SC, MD, AV, and CS: substantial contributions to the acquisition, analysis or interpretation of data, drafting the work or revising it critically for important intellectual content, approval for publication of the content, and agree to be accountable for all aspects of the work in ensuring that questions related to the accuracy or integrity of any part of the work are appropriately investigated and resolved.

## Conflict of Interest

The authors declare that the research was conducted in the absence of any commercial or financial relationships that could be construed as a potential conflict of interest.
