# Supplementary material for: IL-15 and IL15RA in Osteoarthritis: Association With Symptoms and Protease Production, but Not Structural Severity
Source: Front Immunol. 2020 Jul 23;11:1385. doi: 10.3389/fimmu.2020.01385 (PMC7390829; doi:10.3389/fimmu.2020.01385)
Supplement: Supplementary file 1 [file Data_Sheet_1.PDF]

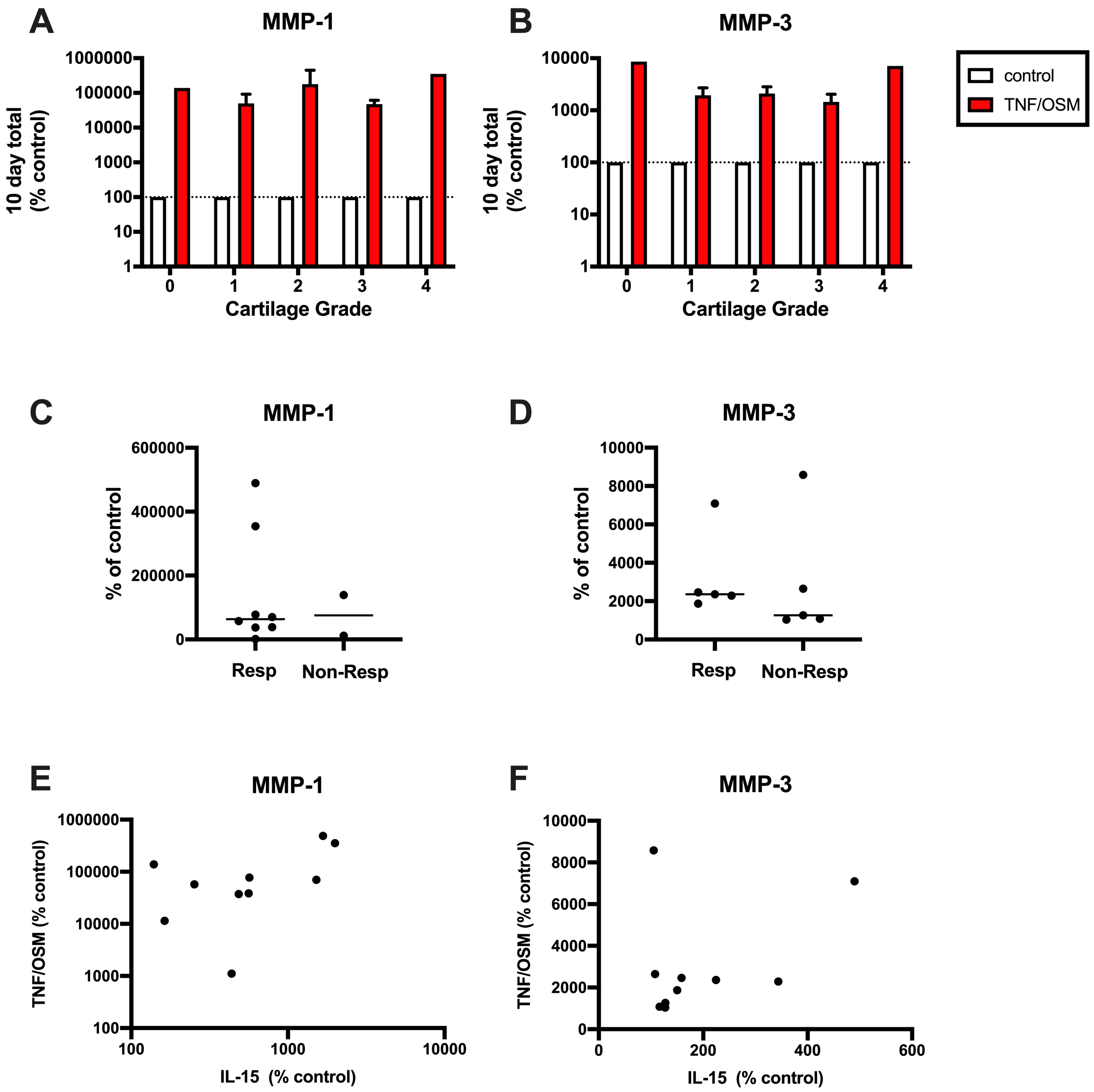

Supplemental Figure 1: Response to TNF/OSM. Articular cartilage explants were exposed to TNF+OSM (100 ng/ml) in vitro for 10 days as described, and protease production measured, as a positive control. Levels of MMP1 (**A,C,E**) and MMP-3 (**B,D,F**) were expressed as percent of the total amount produced in 10 days in unstimulated cultures. TNF/OSM increased release MMP-1 (**A**) and MMP-3 (**B**) in all explant cultures (n=10). As with IL-15, effects were highly variable. However, responses were observed in both normal (grade 0) and degenerative (grades 1-4) specimens. **C&D:** No difference in response to TNF+OSM were observed in IL-15 responders or non-responders (Mann-Whitney $p>0.05$). Similarly, no significant correlation between the responses to IL-15 and TNF+OSM were observed for either MMP-1 (**E:** Spearman $r=0.54$, $p=0.11$) or MMP-3 (**F**: $r=0.05$, $p=0.89$).
